# Measuring malaria endemicity from intense to interrupted transmission

**DOI:** 10.1016/S1473-3099(08)70069-0

**Published:** 2008-04-02

**Authors:** Simon I Hay, David L Smith, Robert W Snow

**Affiliations:** a Malaria Public Health and Epidemiology Group, Centre for Geographic Medicine, Kenya Medical Research Institute/University of Oxford/Wellcome Trust Collaborative Programme, Nairobi, Kenya; b Spatial Ecology and Epidemiology Group, Tinbergen Building, Department of Zoology, University of Oxford, Oxford, UK; c Department of Zoology and Emerging Pathogens Institute, University of Florida, Gainesville, FL, USA; d Centre for Tropical Medicine, University of Oxford, John Radcliffe Hospital, Oxford, UK

## Abstract

The quantification of malaria transmission for the classification of malaria risk has long been a concern for epidemiologists. During the era of the Global Malaria Eradication Programme, measurements of malaria endemicity were institutionalised by their incorporation into rules outlining defined action points for malaria control programmes. We review the historical development of these indices and their contemporary relevance. This is at a time when many malaria-endemic countries are scaling-up their malaria control activities and reconsidering their prospects for elimination. These considerations are also important to an international community that has recently been challenged to revaluate the prospects for malaria eradication.

## Introduction

Malariologists have always sought to grade malaria transmission from intense to interrupted transmission, for conceptual, communication, and ultimately control purposes. The Global Malaria Eradication Programme, coordinated by WHO, focused the research of malariologists and the operational activities of malaria control programmes from around 1950 to 1975.^1^, ^2^, ^3^, ^4^, ^5^, ^6^, ^7^, ^8^, ^9^, ^10^, ^11^ We review a central aspect of the programme's activities: defining criteria for the measurement of malaria and the use of such criteria in guiding malaria control, elimination, and its proposed eradication (panel).^2^, ^12^, ^13^, ^14^, ^15^ The indices selected during the Global Malaria Eradication Programme and their critical values were used to define transitions between activities of malaria control programmes, and also established a common nomenclature for describing malaria risk. The metrics and thresholds achieved a consensus through their widespread application, but were never universally accepted among malaria specialists.

Panel **the nomenclature of disease control, elimination, and eradication**

The terms control, elimination, and eradication need defining. Control is the deliberate reduction of disease incidence to a locally acceptable and manageable level; control must therefore be sustained to maintain the reduction.^16^, ^17^ Elimination is the deliberate reduction of infection incidence to zero in a delimited geographical area; intervention is therefore required to stop re-establishment.^16^, ^17^ Eradication is the permanent global reduction of infection incidence to zero through deliberate efforts; interventions are thus no longer required.^16^, ^17^ The term extinction is not appropriately used in an epidemiological context, because it requires the destruction of the pathogen in nature and the laboratory; equally hard to achieve as to prove.^16^, ^17^ When describing previous work, it is not always possible to use these terms precisely; “certification of eradication” for a country is a non-sequitur, for example; it should be “certification of elimination” and has been so revised in the most recent guidelines.^18^ In these cases the terms are enclosed with inverted commas in the text to highlight the ambiguity.

The collective memory of these measurements and decision rules, their rationale, and experience of their operational performance has waned. Reflection on these considerations is timely because new initiatives have been established to map the global geographical limits of *Plasmodium falciparum* malaria and its endemicity within this range.^19^, ^20^, ^21^ Political commitment to malaria interventions has also been reinvigorated, with many malaria-endemic countries substantially increasing their control activities,^22^, ^23^, ^24^ and others reconsidering the prospects for malaria elimination.^18^, ^25^, ^26^, ^27^, ^28^ Furthermore, the Bill & Melinda Gates Foundation recently issued a challenge to the malaria community to reconsider the prospects for malaria eradication.^29^ The aim of this Review is to evaluate what malaria metrics should now be measured, mapped, and monitored to facilitate malaria control and where possible, its elimination.

## Endemicity and early malariometry

The very etymology of endemic (“in” the population) versus epidemic (“upon” the population) shows the early recognition that the level of a disease, and therefore its character, vary between populations and places. The first method used to quantify malaria endemicity—and thus the first method of malariometry—was introduced in India in 1848 and involved determining the spleen rate (the proportion of a sampled population with palpable enlargement of the spleen^30^) found during a malariometric survey (an investigation of selected age-groups of a randomly sampled population to assess the degree of malarial endemicity in a location).^31^ The term “rate” has unfortunately always been used in the context of malariometric surveys despite the quantity measured being a prevalence. Thus, from the very beginning of malariometry attention was focused on the clinical manifestations of malaria infection in the human population.

How to classify the malaria prevalence estimates obtained by surveys in an epidemiologically meaningful manner was subject to active and prolonged debate. It took over 100 years to reach a consensus that characterised prevalence values from spleen rate surveys as follows: holoendemic more than 75%, hyperendemic 51–75%, mesoendemic 11–50%, and hypoendemic less than 10%, when measured in the 2–9-year-old age-group.^12^ Shortly after this consensus, however, it was suggested that examination of the peripheral blood for asexual malaria parasites by microscopy during malariometric surveys to provide a parasite rate had increased specificity for malaria infection.^13^ However, this process is more invasive for participants, more logistically demanding, and results in a more seasonally variable measure.^31^ Identical names for the endemic levels and their divisions were suggested, with the only difference being that the classification of holoendemic malaria was restricted to infants (those aged less than 1 year) only.^13^ These endemicity classes were put forward as a working hypothesis from expert opinion synthesis of empirical data,^12^, ^31^ but many malaria specialists remained unconvinced of their usefulness. George Macdonald (1903–67) was characteristically vociferous: “The first two of these names [holoendemic and hyperendemic] have come into common use and may well be adopted in a colloquial form, while the last two [mesoendemic and hypoendemic] have not received any general acceptance and do not deserve it”.^32^ Macdonald preferred the stable and unstable classification of malaria endemicity that he derived from a deeper mathematical understanding of the entomological determinants of malaria transmission.^33^

The stable-unstable classification was developed from a dynamic model of transmission that Sir Ronald Ross (1857–1932) used to describe the malaria lifecycle between anopheles and human beings.^34^, ^35^, ^36^, ^37^ Macdonald, in the light of a better understanding of anopheles population biology, was able to express clearly the minimum set of parameters for a simple malaria transmission model (see webappendix).^38^ Importantly, Macdonald then considered the implications of these modelled relations for characterising malaria endemicity,^33^ and showed that the stability of malaria was determined by the average number of feeds that a mosquito takes on a human being during its life (the stability index; formally *a*/−ln*p,* see webappendix for definitions). This vector-based index differentiated stable malaria (insensitive to natural and man-made perturbations, with values more than 2·5) from unstable malaria (very sensitive to climate and very amenable to control, with values less than 0·5).^33^ Intermediate stability was designated between these extremes. Macdonald deemed this distinction fundamental and stated that “other classifications should be subordinate to this”.^33^ The stable-unstable stratification has the virtue of being able to classify malaria endemicity entirely from information on the bionomics of locally dominant anopheles vectors.^2^ Like other vector-based malaria metrics,^39^, ^40^, ^41^, ^42^ the stable-unstable concept is rarely implemented and is used less precisely than Macdonald would have condoned. The reasons for the paucity of vector-based indices are the technical complexity of obtaining entomological-based metrics, ethical concerns related to exposing human beings to malaria infection, and measurement error issues.^41^, ^42^, ^43^, ^44^

Regardless of the theoretical range of endemicity measurements that could be used, host-based assessments of malaria prevalence through malariometric surveys dominate the formal and informal contemporary literature,^20^ and are hence considered here in detail.

## The statistics of prevalence

The prevalence of any condition is measured from a sample of a reasonably homogeneous population so that its precision can depend on the sample size and the amount of the disease.^45^, ^46^, ^47^ The confidence we can place in an estimate of prevalence will decrease as the numbers sampled become smaller or as the disease becomes rarer. Detailed guidance on statistical sampling in malariometric surveys^48^, ^49^, ^50^ was outlined for malaria control personnel as part of the mentoring activities of the Global Malaria Eradication Programme.^1^, ^2^, ^3^, ^4^, ^5^, ^6^, ^7^, ^8^, ^9^, ^10^, ^11^ It is clear that as the reliability of a malariometric survey diminishes with declining prevalence, the population sampled must increase for a specified level of confidence in an estimate to be maintained. Since obtaining blood from people is not without cost (personal and programmatic), there comes a point when measuring prevalence is not advisable. This was defined operationally during the Global Malaria Eradication Programme: “As soon as the general volume of malaria has been reduced to any considerable extent, the indices furnished by malariometric surveys are no longer sensitive enough to measure further progress…Analysis of evaluation data from eradication programmes as well as closer observations in the field have shown that the point at which malariometric surveys cease to be sufficiently sensitive is reached when parasite rates have dropped to a level of between 1% and 3%”,^51^ although other experts have since argued for a greater flexibility in these parasite rate levels.^52^ Therefore, when malaria prevalence dropped below these levels, alternative measures were required. These measures have usually been obtained by looking at the rate of diagnostically verified clinical malaria in a population; its true incidence.

## Measuring incidence

The measurement of malaria incidence requires every suspected malaria case to be diagnosed through a comprehensive surveillance system comprising passive case detection (examination of suspected, usually febrile cases presenting routinely to any point of the health service), supplemented by active case detection (examination of fever cases sought through home visits at regular intervals).^15^, ^46^ The results are usually expressed as an annual parasite incidence (API) per 1000 of the population of the administrative area it represents. During the Global Malaria Eradication Programme, the API was only deemed valid if the annual blood examination rate (ABER)—the proportion of the target population examined—exceeded 10%.^1^, ^2^, ^5^ The other metric often presented in this surveillance trio is the slide positivity rate (SPR), the percentage of examined slides found positive. These surveillance indices are related as follows: API=(ABER*SPR)/10.^53^ The division by ten is necessary because API is expressed per 1000 and the other terms per 100.

## The Global Malaria Eradication Programme plan of intervention

**Figure 1:**
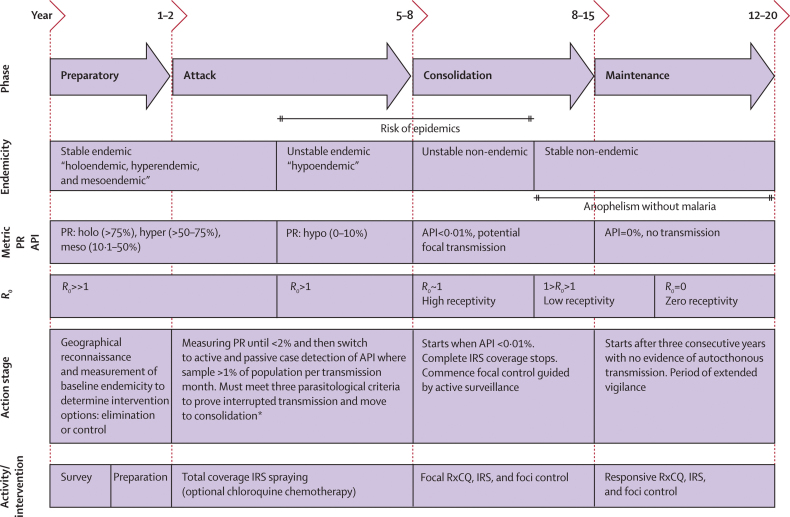
Malaria endemicity and classification, and timelines for action phases of the Global Malaria Eradication Programme The scheme derives details of the phase, approximate timings, action stage, and activity of the Global Malaria Eradication Programme^1^, ^2^, ^3^, ^5^ and integrates these with malaria endemicity classifications defined by the host prevalence,^12^, ^13^ vector stability indices,^33^ and the basic reproductive number (see webappendix).^46^ The year row presents an estimate of the duration of each phase (optimistic scenario–pessimistic scenario). API=annual parasite incidence. IRS=indoor residual spraying. PR=parasite rate. *R*_0_=basic reproductive number. RxCQ=radical treatment with chloroquine. *See table for parasitological criteria.

The measurement of the parasite rate and the API had crucial roles in the Global Malaria Eradication Programme and together helped define the transitions between the four phases of intervention: preparatory, attack, consolidation, and maintenance.^1^, ^2^, ^3^, ^5^ We have attempted to reconcile the measurements and transition points used in the programme with the epidemiological classifications outlined previously (figure 1). It is worth noting that in the Global Malaria Eradication Programme literature, the parasite species was not routinely defined in relation to these indices, since the goal was to eradicate all *Plasmodium* species that infect human beings.

The main objective of the Global Malaria Eradication Programme was to interrupt malaria transmission through a time-limited, geographically comprehensive, indoor residual spraying campaign of sufficient duration to eliminate the parasite reservoir, while minimising the time for the development of insecticide resistance (usually suggested as occurring after 3–4 years).^1^, ^2^, ^3^, ^5^

Malaria parasite rate surveys were recommended during the first phase of the programme to evaluate whether the endemic level precluded the prospect of cessation of transmission in this 3–4-year timeframe: to a good approximation the holoendemic areas of Africa.^54^ The exact prevalence levels for defining this important division seem to have been avoided, however.^1^, ^2^, ^3^, ^5^ If “eradication” was deemed feasible, a survey of the homes to be sprayed was undertaken and planning for indoor residual spraying implemented. Spraying usually started within 1–2 years and the declining parasite rate was used to monitor progress and to verify the interruption of transmission.^14^

The attack phase was usually estimated to take 3–4 years; 1–1·5 years for the interruption of transmission, plus 1–1·5 years or 2–2·5 years for the disappearance of the *P falciparum* and *Plasmodium vivax* parasite reservoirs in human beings, respectively. Comprehensive surveillance was initiated when the parasite rate fell below 2%, but in reality was often initiated 2 years after the onset of the attack phase (on the assumption of success in interrupting transmission), since it took control programmes time to establish the necessary surveillance infrastructure. An imminent move to the consolidation phase was indicated when the parasite rate was 2% or less and three other parasitological criteria were met^1^, ^2^, ^3^, ^5^ (figure 1 and table). The criteria that were necessary to confirm the interruption of transmission were suggested by Macdonald^14^ and later endorsed by WHO^55^ as: (1) the parasite rate in populations greater than 3 years of age decreased by 78% in successive years (a ratio of 1:0·22 per year); (2) the parasite rate in those born after the onset of the programme was zero; and (3) the prevalence of heavy infection (defined as more than 1000 parasites per μL of blood) was zero. Less strict criteria were necessary for the interruption of transmission in a “reasonable” time (figure 1 and table).^55^

**Table:** Parasitological criteria required to prove interrupted transmission for transition from the attack to consolidation phases of malaria control

	**Complete interruption of transmission**	**Maximum allowed with prospects for interruption of transmission within a reasonable time**
PR of age-group above 3 years	PR_t+1_≤0·16PR	PR_t+1_≤0·22PR
Infant PR as percentage of the total population PR	0%	10%
Prevalence of heavy infection* percentage of the total population PR	0%	7%

PR=parasite rate. PR_t+1_=parasite rate in the following year.

* >1000 parasites per μL blood.

When the API was less than 0·01%, the consolidation phase started and comprehensive indoor residual spraying stopped.^56^ Originally this value was set higher (API 0·05% or less),^57^ but was reduced when experience showed that national malaria “eradication” programmes often overestimated the comprehensiveness of their surveillance, meaning that outbreaks were common after the cessation of indoor residual spraying at this API level. The consolidation phase maintained a targeted control component, guided by active case detection to eliminate residual foci in human reservoirs and the remaining “islands” of transmission in the environment. During the Global Malaria Eradication Programme, suspected malaria cases were treated presumptively (without awaiting the result of microscopic diagnosis) and radically (with an effective drug or drug combination with the intention of killing all blood and liver-stage parasites to prevent any possibility of relapse). The duration of the consolidation phase was highly variable, depending as much on the longevity of the political commitment as on the general epidemiological context.^1^, ^2^, ^3^, ^4^, ^5^, ^6^, ^7^, ^8^, ^9^, ^10^, ^11^

Movement to the maintenance phase was initiated after 3 years without local transmission.^58^ To prove this status, a high level of surveillance was required with evidence that every new case could be classified as: (1) imported (a malaria case whose origin could be traced to an area of transmission outside the country in which it was diagnosed); (2) relapsing (renewed clinical symptoms and/or parasitaemia resulting from an original infection because of the survival of hypnozoites [thus specific to *P vivax* and *Plasmodium ovale*] for a period longer than the normal periodicity of paroxysms); (3) induced (a malaria case resulting from any form of parenteral inoculation); or (4) introduced (a first-generation case of locally transmitted malaria following an imported case).^31^, ^58^ The criteria for movement to the maintenance phase were somewhat ambiguously extended to include an assessment of the likelihood of maintaining “eradication” in the later stages of the Global Malaria Eradication Programme.^56^

The maintenance phase involved the introduction of vigilance measures by the public-health service, rather than the specialised malaria “eradication” teams. This phase required assessments of the vulnerability (the probability of reintroduction based on an area's proximity to other malarious areas and the flux of infected human beings or anopheles mosquitoes between the two) and receptivity (the natural endemic level) of an area, which together defined its malariogenic potential. We do not comment on the feasibility of maintaining elimination because this has been comprehensively reviewed elsewhere,^3^, ^59^ but emphasise that two broad types of failures were identified during the Global Malaria Eradication Programme. First, areas where the endemic level of malaria was such that transmission was never interrupted^60^, ^61^, ^62^, ^63^ and second, areas where transmission was interrupted but could not be maintained because the health-care infrastructure could not support the required level of vigilance.^3^, ^59^, ^64^, ^65^ It is also worth noting that guidance on how to measure the concepts of vulnerability and receptivity was never explicit, despite their central importance.

The certification of malaria “eradication” by WHO had its own set of standards^55^, ^57^, ^66^, ^67^ and areas reaching certification status were entered into a register following inspection by a WHO delegation. A general pessimism surrounds many reviews of the Global Malaria Eradication Programme,^7^, ^8^, ^9^, ^10^, ^11^, ^59^ but it should be remembered that 24 countries eliminated malaria, partly as a result of its activities. The first of these was northern Venezuela (June, 1961) and the last Singapore (November, 1982).^5^, ^26^ Furthermore, despite the replacement of the programme with the Global Malaria Control Strategy,^68^ many countries still pursue the goal of malaria elimination.^25^, ^26^, ^27^, ^28^ In 2007, the United Arab Emirates became the first country to receive official WHO certification of malaria elimination in the post-Global Malaria Eradication Programme era.^18^, ^69^

What is important to understand, for those countries currently scaling-up control activities or targeting elimination, is do the metrics and action points mentioned above remain feasible, plausible, and useful today? To explore this question further, and before looking at the modern context of malaria control, we have examined the predictions of simple mathematical models (see webappendix). It is also important to note that *P vivax* has biological, clinical, and epidemiological differences that make it more difficult to measure, model, and control.^70^, ^71^, ^72^ By contrast with the Global Malaria Eradication Programme, all further considerations are specific to *P falciparum* malaria.

## The revised context

Despite the long-term vision of the Millennium Development Goals,^73^ national malaria control programmes, regional offices of WHO, and international development agencies are constrained by the periodic (usually quinquennial) allocation of funding. It is therefore pragmatic to consider 5-year funding cycles that provide a country with opportunities for staged impacts along the path towards its longer term goals.

By contrast with the Global Malaria Eradication Programme, diverse interventions are now used and most malaria-endemic countries have developed their own comprehensive intervention plans. It is assumed that policy makers in malaria-endemic countries would wish to implement a combined set of widespread interventions,^74^ including (but not limited to^75^, ^76^) the free distribution of insecticide-treated bednets,^77^, ^78^, ^79^, ^80^ indoor residual spraying,^81^, ^82^, ^83^ prompt and effective radical treatment with artemisinin-based combination therapy,^81^, ^84^ and intermittent preventive treatment (a curative dose of an antimalarial drug given at fixed times in high-risk groups such as pregnant women and infants, regardless of infection).^85^, ^86^ Environmental management and larval control might also be cost effective, particularly in high human population-density urban areas.^41^, ^87^, ^88^, ^89^ Although each of these interventions will substantially affect transmission, the combination of their effects and how to optimise them is largely unknown. How can we use the evidence available to evaluate optimal control choices and their potential impact during the typical funding cycle? This evaluation is now complicated by the scaling-up of malaria control. Everyone is susceptible to clinical malaria, albeit with different levels of risk, but transmission is reduced when insecticide-treated bednets are used universally because the chances of the mosquito vectors becoming infected and living long enough to become infective and bite human beings are reduced. Insecticide-treated bednet distribution programmes should aim to cover the whole population, not just the most vulnerable.^90^ Classic intervention trials are not possible within the context of universal insecticide-treated bednet coverage, since people cannot be denied access to them. The impact of combinations of interventions will now need to be assessed (and modelled) through carefully designed surveillance trials, where the incremental effects of intervention modes over universal bednet coverage alone are tested.

Finally, the current status of global malaria surveillance is a cause for concern.^91^, ^92^, ^93^, ^94^ Very few contemporary studies have tested the reliability of national case surveillance activities^91^, ^95^ and this has obvious but unpredictable operational ramifications. Conversely, the past 5 years have seen a substantial increase in the use of malariometric surveys in national indicator surveys^70^, ^96^, ^97^, ^98^ that provide detailed spatial epidemiological information for specific countries. A further challenge when establishing revised criteria, therefore, is to recognise and consider these problems and trends in malariometry.

## A revised scheme

**Figure 2:**
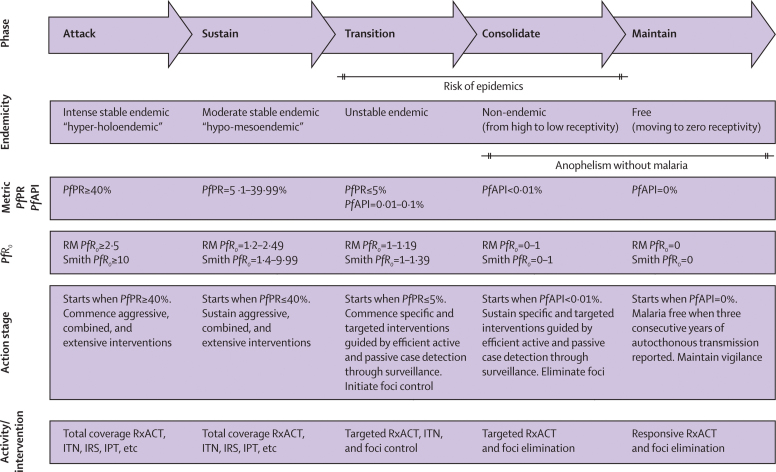
Malaria endemicity and classification, and the mapping criteria of the Malaria Atlas Project^19^ The *PfR*_0_ (*P falciparum* basic reproductive number) values are calculated using the RM (Ross-Macdonald) and Smith transmission models (see webappendix for full details). IRS=indoor residual spraying. IPT=intermittent preventive therapy. ITN=insecticide-treated net. *Pf*API=*P falciparum* annual parasite incidence. *Pf*PR=*P falciparum* parasite rate. RxACT=radical treatment with artemisinin-combination therapy.

Refinements in the way that malaria risk is measured and mapped are suggested, along with some practical considerations, in figure 2. For continuity, these changes are related to classic endemic divisions, as shown for the Global Malaria Eradication Programme phases (figure 1). A deliberate effort has been made to diminish the clear divisions between the now five stages of intervention: attack, sustain, transition, consolidate, and maintain. We do not make guesses about the duration of intervention phases, since the modelling framework to support these speculations requires further work. Rather, we view them pragmatically as steps of 5-year funding cycles that are implemented sequentially over one to two decades to achieve a long-term goal. This extended timeframe is based on the experience of the Global Malaria Eradication Programme, which showed that the time required to move from the attack to the maintenance phase (figure 1) was highly variable between countries^1^, ^2^, ^3^, ^4^, ^5^, ^6^, ^7^, ^8^, ^9^, ^10^, ^11^ and sometimes in the order of decades, even when starting from low endemicity.^99^, ^100^, ^101^, ^102^, ^103^, ^104^, ^105^, ^106^, ^107^ A suggested scheme for monitoring the steady progress through these steps is outlined (figure 2). It is emphasised that figure 2 does not imply that that all areas can migrate successfully from attack to maintenance only that the trajectory will be similar: the feasibility of this transition will be mitigated by locally variable epidemiological, environmental, logistical, and geopolitical factors.

The attack phase starts in all areas where the *P falciparum* parasite rate (*Pf*PR) is 40% or more; a threshold from which theory predicts it is unlikely to interrupt the transmission of malaria with insecticide-treated bednets alone (see webappendix). In these areas, the attack phase might begin with a combination of interventions applied nearly universally in a population. Reductions in clinical malaria are expected following high coverage, but a drop in the incidence of clinical malaria is not adequate indication that malaria transmission has been sufficiently reduced. Theoretical considerations and practical experience suggest that the progress of the attack phase should be measured by repeated *Pf*PR-based malariometric surveys, as was done during the Global Malaria Eradication Programme. Monitoring impact with malariometric surveys is also consistent with the trend for incorporating parasite rate data collection in national indicator surveys.^70^, ^96^, ^97^, ^98^ Since the Global Malaria Eradication Programme era, further work has revealed a range of factors that can affect the precision with which *Pf*PR is measured, including the performance of microscopists^108^, ^109^ and the timing of the *Pf*PR measurement during the course of an infection.^110^ These measurement error issues remain important, but as rapid diagnostic tests become increasingly widespread,^111^, ^112^ the logistical ease of malariometric surveys will increase. Previous work has indicated that sampling the 2–10-year age-group is optimal^113^ and that age standardisation techniques can be applied to help compare malariometric surveys done in different age cohorts. Even with continuous, combined interventions deployed effectively on a large scale, it may be difficult to move some very high transmission areas to an endemic level that would indicate a transition to the sustain phase (*Pf*PR less than 40%).

By contrast with the Global Malaria Eradication Programme, the intervention activities continue during transition from the attack to the sustain phase. Impact continues to be measured with malariometric surveys until *Pf*PR is 5% or less. We have raised the level of a shift to surveillance from the 2–3% level used by the Global Malaria Eradication Programme because, as modelling shows (see webappendix), the information provided for control is minimal (all levels below *Pf*PR 10% imply extremely low transmission) and the error and numbers required for reliable surveys is maximal. As the *Pf*PR falls below the 10% level, substantial effort should be invested in improving the rigour and depth of active and passive case detection.

When *Pf*PR is 5% or less, the transition phase is reached. In addition to malariometric surveys, complementary measurements of *Pf*API, over a series of 3 years, should also show *Pf*API below 0·1% per year. The pairing of these metrics at these levels is justified by the results of the largest ever simultaneous review of the global *Pf*API and *Pf*PR data.^21^ This stage represents an important transition conceptually and operationally, where active and passive case detection for clinical malaria become the dominant measurement tools. The stage is central to informing the direction of intensified control activity, targeted at the last foci of transmission.

The movement to the consolidation phase is signalled when *Pf*API is less than 0·01% per year and is informed by experience of the Global Malaria Eradication Programme.^58^ It may also be prudent to add an additional criterion to sustain this level over a period of years, to ensure that there have been no lapses in surveillance.

The movement to the maintenance phase is heralded when *Pf*API equals 0%. The rigour with which countries undertake these final surveillance and audit stages should exceed those suggested during the Global Malaria Eradication Programme, exemplified in recent times by the malaria control programme in the United Arab Emirates.^18^, ^69^ This increased enforcement is needed because the risks of the reintroduction of imported malaria and malaria vectors has increased with the expansion of global trade and travel.^114^, ^115^, ^116^ In the past, confirming the origin of any *P falciparum* cases found during the maintenance phase and any outbreaks after a country had been declared malaria free, was achieved through standard epidemiological investigation and serology.^117^ Today, whether these cases represent clusters of local transmission can be reliably established by genotyping.^118^

## Discussion

In a time of renewed political and national commitment to malaria control, there is much that we still do not know. Modelling the potential impact of existing combinations of interventions and how these can be best combined is an obvious need. This information would enable a further control-related classification of endemicity above the *Pf*PR 40% level. When considering only the most conservative modelling framework (see webappendix), a much larger proportion of the world than is traditionally considered could be suitable for elimination.^1^, ^2^, ^3^, ^4^, ^5^, ^6^, ^7^, ^8^, ^9^, ^10^, ^11^ Recent companion work shows that nearly 1 billion (42%) of the 2·37 billion people at risk of malaria in 2007 live in areas of unstable transmission (*Pf*API less than 0·01% per year), from which elimination is theoretically feasible on epidemiological grounds.^21^ Substantial modelling work is required to investigate the prospects for control and elimination in areas of stable transmission and mapping work is required to show where these areas are located.

The time taken for interventions to achieve their desired effect is also an area of uncertainty and is an extension of this modelling at the high-transmission end of the spectrum. Even without this caveat, we are mindful here of the advice of Paul F Russell (1894–1983) that “Time more than money and continuity more than perfection—these must be the mottoes guiding malaria control in the tropics”,^119^ and reiterate that decades of work are required to achieve elimination, even from a modest baseline endemicity.^99^, ^100^, ^101^, ^102^, ^103^, ^104^, ^105^, ^106^, ^107^ In principle we can feel optimistic about the theory of elimination, but this needs to be tempered by the realities of practice.^1^, ^2^, ^3^, ^4^, ^5^, ^6^, ^7^, ^8^, ^9^, ^10^, ^11^ Additionally, the mathematics of low-level *P falciparum* malaria transmission is not adequately addressed using these classic and deterministic modelling frameworks. Although these concerns were not completely ignored historically,^120^, ^121^, ^122^, ^123^ and more recent contributions have been made,^124^ the modelling of low-level malaria transmission for the purposes of optimising elimination merits attention. As infection becomes rare, it might be more efficient to replace campaign-style malaria control with a focus on people who have clinical malaria and their neighbours who may be infected asymptomatically.^76^ The crucial process is to find and eliminate the largely invisible reservoir of parasites in human beings. Understanding transmission in these environments is essential and demands stochastic models that deal with rare infections in individuals in an explicitly spatial context.

The heterogeneity in transmission, necessary in the newer malaria transmission models (see webappendix) to more realistically describe the inter-relations between *Pf*PR, *Pf*EIR (*P falciparum* entomological inoculation rate) and *PfR*_0_ (*P falciparum* basic reproductive number),^125^, ^126^ is likely to be important in malaria control^127^ and elimination. Whether these sources of heterogeneity can be identified, mapped, and targeted to maximise the effect of interventions remains unclear. A large remaining topic for research is identifying the human and vector-based contributions to this transmission heterogeneity.^125^, ^126^

Quantifying vulnerability and receptivity was never done rigorously during the Global Malaria Eradication Programme (although it is amenable through the modelling framework, see webappendix) and is fundamental to those countries or regions wishing to maintain a malaria-free status. Useful guidelines on aspects of these activities have been released^18^, ^128^, ^129^ and although they represent thorough reviews, they have yet to fully embrace some of the opportunities offered by high-resolution satellite imagery renderings of the Earth (eg, Google Earth) or the increasing ability to quantify and evaluate the epidemiological impact of human transport systems.^114^, ^115^, ^116^ Moreover, serological tests are now available that can assess the history of individual infections and help to verify the interruption of transmission, especially when age-stratified^130^, ^131^, ^132^, ^133^ (as first demonstrated in Mauritius^134^), and to establish the provenance of the natural parasite reservoir versus imported cases.^135^ If these serological histories could be inferred from the blood samples collected in national indicator cluster surveys with rapid diagnostic tests, then the epidemiological content of investigations would be greatly enhanced. Additionally, pinpointing sampling clusters with hand-held global positioning systems is extremely valuable for mapping.

Adopting a malaria classification of *Pf*PR guided by the modelled feasibility of control and elimination is a primary goal for the Malaria Atlas Project alongside efforts to map the global limits of malaria and its endemicity within this range.^19^, ^20^, ^21^ Together these data may provide a platform to map the likely impact of interventions and the successive 5-year steps required to realise national plans. It will soon be possible to document what proportion of the global population is at risk of *P falciparum* malaria, to what level of endemicity they are exposed, and therefore the number of people living in each intervention stage. It will then be possible to audit the cost and time required to move these populations towards endemic levels compatible with sustainable control and where feasible, elimination.

## Conclusions

Despite metrics being useful guides, countries that are implementing malaria control interventions should include flexibility and local knowledge in the application of any decision criteria, because “In the study of malaria problems and in the formulation of control programmes, action based on generalisations is likely to be followed by the most disastrous consequences. It has been well said that the most hazardous of human tendencies is the drawing of general conclusions from limited experience, and in no instance is it more applicable than in the planning of malaria control measures”.^136^ There is still much to do in terms of assembling relevant data,^19^, ^20^, ^21^ addressing these with appropriate models, and making the maps to help refine these generalisations to more specific action plans at regional, national, and subnational levels.

## Search strategy and selection criteria

Data for this Review were identified by searches of PubMed, ISI Web of Science, the WHO library, and bibliographies of retrieved articles, and through suggestions of reviewers (formal and informal). We used the following Boolean search statement: “malaria” AND (“eradication” OR “elimination”), “malaria” AND (“control” OR “campaign”), “malaria” AND (“survey” OR “metric” OR “measure”). Articles in English, French, and Spanish were selected and no date restrictions were applied to the searches.

## Web Extra Material

Webappendix
