# Supplementary material for: Measuring malaria endemicity from intense to interrupted transmission
Source: Lancet Infect Dis. 2008 Apr 2;8(6):369–78. doi: 10.1016/S1473-3099(08)70069-0 (PMC2653619; doi:10.1016/S1473-3099(08)70069-0)
Supplement: Webappendix [file mmc1.pdf]

Measuring malaria endemicity from intense to interrupted transmission

Correspondence to:
Dr Simon Iain Hay, Malaria Public Health and Epidemiology Group, Centre for Geographic Medicine, Kenya Medical Research Institute/University of Oxford/Wellcome Trust Collaborative Programme, Kenyatta National Hospital Grounds, PO Box 43640-00100, Nairobi, Kenya. Tel +254 20 2715160; fax +254 20 2711673; **shay@nairobi.kemri-wellcome.org**

Mathematical modelling

For more than a century of malaria control, the modelling framework has been central to devising epidemiologically informed intervention strategies and the means by which to measure their impact.[1,2] George Macdonald (1903–67), more than any other malariologist, used models to influence policy during the Global Malaria Eradication Programme,[3] which led to their further development and testing.[4–10] The models have since been further refined[9,11–15] and predict relations between malaria metrics that have implications for the classification of malaria risk. These implications are explored below.

The basic Ross-Macdonald model of malaria transmission[4,16–19] using the notation commonly used by malariologists is as follows:

$$R_0=\frac{ma^2bcp^n}{r(-\ln p)}$$

where *m* is the ratio of anophelines to human beings (number of anophelines/number of human beings), *a* the human biting rate (number of bites on a human being per anopheline per day), *b* the transmission efficiency of infected anopheline to human being, *c* the transmission efficiency of infected human being to anopheline, *p* the proportion of anophelines surviving 1 day, *n* the duration of sporogony (days)—the process of parasite development occurring in the anopheles mosquito that follows sexual union of gametes and ends with the formation of infective sporozoites—and *r* the rate of recovery of the human being from infection (days), so that $1/r$ is the human infectious period. Put together correctly, these parameters define the classic, steady-state version of the basic reproductive number for malaria, R_0, the expected number of hosts who would be infected by a single infectious person, introduced into an otherwise naive population, after one generation of the parasite. The R_0 is thus a threshold concept; if <1 a disease will die out and if >1 it will increase. The greater the positive value the more rapid its rate of increase. The R_0 concept has been used to help classify malaria endemicity[20] and this scheme has additionally been incorporated into figure 1 and figure 2 of the main text. These models are applicable to all malaria and generalised to a hypothetical anopheles vector. They are considered specifically with respect to *Plasmodium falciparum* transmission below.

Previous work[13–15] has shown that the Ross-Macdonald model predicts well-defined three-way relations between *Pf*PR (*P falciparum* parasite rate), *Pf*EIR (ε) (*P falciparum* entomological inoculation rate), and PfR_0 (*P falciparum* basic reproductive number) at the steady state:

$$PfPR=\frac{b\varepsilon}{r+b\varepsilon}=\frac{PfR_0-1}{PfR_0+cs}$$

$$\frac{b}{r}\varepsilon=\frac{PfPR}{1-PfPR}=\frac{PfR_0-1}{1+cs}$$

$$PfR_0=\frac{1+csPfPR}{1-PfPR}=1+\frac{b}{r}\varepsilon\,(1+cs)$$

All notation is the same as in the first Ross-Macdonald equation, but for simplicity we express the stability index, *s*, rather than $a/\text{-}\ln p$. If a control programme reduces PfR_0 uniformly by a factor, *F*, then *P falciparum* malaria will be eliminated everywhere that $R_0<F$, or where (note the formulae give daily ε if the units of *r* are days):

$$PfPR<\frac{F-1}{F+cs}$$

$$\frac{b}{r}\varepsilon<\frac{F-1}{1+cs}$$

For example, if $F=10$ (a 90% reduction in transmission), then malaria will be eliminated wherever *Pf*PR<80%, or equivalently a *Pf*EIR<10 (assuming $b=0\cdot8$, $1/r=200$, $c=0\cdot5$, and $s=1$). Obviously, these formulae ignore imported malaria, heterogeneous biting, and many other factors known to be of importance for malaria transmission but are nevertheless useful to structure thinking.

Mathematical models and the relations between malaria metrics

The prediction of the ubiquitous Ross-Macdonald formulations[4,16–19] can be compared graphically with more recent revisions[14] to show the relation between the host-based measures of prevalence (the *Pf*PR) and the vector-based *Pf*EIR, the number of *P falciparum* infective bites per human being, per unit time (webfigure 1).[21,22]

The relation between *Pf*PR and *Pf*EIR predicted by the Ross-Macdonald theory shows that *Pf*PR is very sensitive to small increases in *Pf*EIR at low transmission intensity and insensitive where transmission is high (webfigure 1). The *Pf*PR is therefore an excellent measure of malaria endemicity before the relation saturates at holoendemic transmission levels, where theoretically, *Pf*EIR would be a more valuable guide, although for mainly logistic reasons (see main text) it is infrequently sampled.[21,22]

This *Pf*PR-*Pf*EIR relation has also been corroborated empirically[21,24] using simultaneous measures of *Pf*PR and *Pf*EIR in communities. These in turn have further helped

reveal the importance of incorporating heterogeneity into malaria transmission models.[14,15] Heterogeneity in transmission (irrespective of cause) intensifies the *Pf*PR-*Pf*EIR relation at low endemicity (since bites are more focused on infected and infectious individuals: super-spreaders), but this effect becomes increasingly moderated with higher prevalence (these same bites are concentrated on already infected individuals: super-absorbers; webfigure 1). There is little argument therefore that malariometric surveys remain an appropriate choice for assessing the impact of control measures.

It is also clear that, under both modelling frameworks, any value of *Pf*PR≤10% is significantly below a *Pf*EIR of one infectious bite per year (webfigure 1) and thus constitutes a low and operationally uniform transmission stratum. The decision about when to stop malariometric surveys and move to surveillance is not informed by theory but is guided mainly by the point where the sample sizes required become operationally prohibitive (see main text).

It is possible to examine the modelled expectation of the relation between *Pf*PR and the basic reproductive number, R_0, for *P falciparum* malaria, in the same way that we have detailed the relation between *Pf*PR and *Pf*EIR (webfigure 2). The PfR_0 is the perfect metric for evaluating control and elimination feasibility, because it was derived from theory for specifically that purpose[26] (see above) and is discussed in the following section.

Mathematical models and the feasibility of control

Since the time of the Global Malaria Eradication Programme, malaria control programmes have become increasingly plural in the range of interventions that they consider[27] and are generally more cognisant of the fact that their optimal mix should be stratified by ecological, entomological, and sociological settings.[28–30] Despite the diversity of interventions available, those which are routinely taken to scale are few and the deployment of insecticide-treated bednets is currently the most widespread.[31–36] It has been shown by many field trials[37] and recent theory[38] that a ten-fold reduction in *Pf*EIR can be achieved with a 45–75% coverage of bednets (ownership multiplied by use), depending on the local vector species. What effect might this order of reduction have on our malaria metrics and how does this relate to classically defined endemicity levels?

The immediate impact of insecticide-treated bednets on PfR_0 are directly proportional to the immediate reductions in *Pf*EIR and to the baseline *Pf*PR. PfR_0 is directly proportional to vectorial capacity, *V*. Mathematical theory predicts that *Pf*EIR will change in two phases after starting vector control: $dPfR_0/dt \approx dPfEIR/dt = dV/dt * PfPR + V dPfPR/dt$.[38] The largest and most immediate reductions in *Pf*EIR are caused by reductions in the vector populations and changes in their age composition, as measured by vectorial capacity: but on fast time scales, $dPfPR/dt \approx 0$. Thus, the immediate changes in *Pf*EIR are caused by changes in vectorial capacity scaled by *Pf*PR, which translate directly into changes in PfR_0. Later, *Pf*EIR will fall even further because of feedback with *Pf*EIR reducing the proportion of people who are infectious to mosquitoes. In places where PfR_0 is high, the slower changes are also small because *Pf*PR is relatively insensitive to the initial changes in *Pf*EIR. Where PfR_0 is low enough and transmission is interrupted by vector control, elimination may follow after a few years. These principles apply to any sustained reductions in vector populations achieved through vector control, whether through insecticide-treated bednets, indoor residual spraying, or some other means. The actual reductions achieved through vector control vary, depending on the vector and the coverage achieved.[38] Recent studies have typically not measured reductions in *Pf*EIR as an intermediate endpoint, so there has been very little entomological data to evaluate. Where measured,

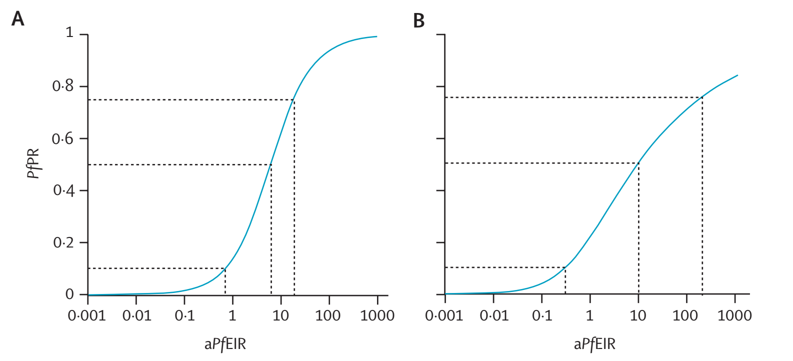

***Webfigure 1*: The inter-relations between the *P falciparum* parasite rate (*Pf*PR) and annual *P falciparum* entomological inoculation rate (a*Pf*EIR)**

Calculated using (A) the Ross-Macdonald[4,16] and (B) the Smith[14] modelling frameworks. The dotted lines mark the prevalence levels that transition between the canonical endemicity classes.[23]

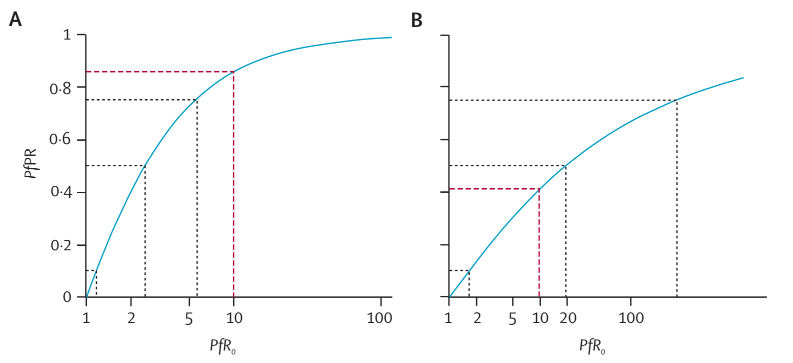

***Webfigure 2*: The inter-relations between the *P falciparum* parasite rate (*Pf*PR) and the *P falciparum* basic reproductive number (PfR_0)**

Calculated using (A) the Ross-Macdonald[4,16] and (B) the Smith[14] modelling frameworks. The dotted lines (black) mark the prevalence levels that transition between the canonical endemicity classes.[23] The dotted lines (red) show the *Pf*PR level that can be completely controlled with a ten-fold reduction in PfR_0. These relations are modelled simplifications because control effects are not instantaneous and will take time to manifest as changes in community prevalence caused by the duration of *P falciparum* infections[25] and other factors (see text), but they are useful guides.

insecticide-treated bednets and indoor residual spraying have achieved factor of ten reductions,[39,40] although much larger decreases were achieved during the Global Malaria Eradication Programme.[41–44]

The first important observation is that the classic *Pf*PR-based divisions of malaria endemicity[23] have no clear relation with PfR_0 and thus to the feasibility of control or elimination. It is clear from these considerations that there is a crucial need to investigate retrospectively, through the plethora of documented control and "eradication" attempts, the performance of mathematical models at predicting the success of interventions in highly endemic areas. Second, the Ross-Macdonald models show that almost the full *Pf*PR prevalence spectrum (0–90%) is spanned in a range of $R_0<10$ (webfigure 2). The theoretical inferences one might draw from these models on the ease of control are difficult to reconcile with historical examples of very determined, comprehensive, and mixed intervention strategies that did not achieve an interruption of transmission in highly endemic areas.[41–44] In other words, it seems unlikely that such vigorous control didn't achieve a ten-fold reduction in PfR_0, which the Ross-Macdonald models predict should end transmission even at holoendemic levels. The more recent Smith models[14] that include transmission heterogeneity indicate that the R_0 range is more plausibly extended across the *Pf*PR transmission spectrum, so that holoendemic malaria with *Pf*PR≥75% equates to $PfR_0\geq150$ (webfigure 2). The differences predicted by these models obviously impact considerably on any evaluation of the feasibility of malaria control by endemic level. Even if the more conservative modern framework[14] is adopted and a ten-fold reduction in PfR_0 assumed, all populations with a natural baseline *Pf*PR≤40% would be able to eliminate malaria by the scaling-up of insecticide-treated bednets (webfigure 2).

These mathematical models therefore offer further scope for guided hypotheses on the feasibility of control and/or elimination, when combined with additional evidence on the impact of existing interventions. These are expanded further in the main text.

References

1 Nájera JA. Epidemiology in the strategies for malaria control. *Parassitologia* 2000; **42:** 9–24.

2 McKenzie FE, Samba EM. The role of mathematical modeling in evidence-based malaria control. *Am J Trop Med Hyg* 2004; **71:** 94–96.

3 Macdonald G. Theory of the eradication of malaria. *Bull World Health Organ* 1956; **15:** 369–87.

4 Macdonald G. The epidemiology and control of malaria. London: Oxford University Press, 1957.

5 Nájera JA. A critical review of the field application of a mathematical model of malaria eradication. *Bull World Health Organ* 1974; **50:** 449–57.

6 Bruce-Chwatt LJ. Swellengrebel oration: mathematical models in the epidemiology and control of malaria. *Trop Geogr Med* 1976; **28:** 1–8.

7 Molineaux L, Gramiccia G. The mathematical model of transmission. In: The Garki Project. Research on the epidemiology and control of malaria in the Sudan savanna of West Africa. Geneva: World Health Organization, 1980: 261–88.

8 Molineaux L, Gramiccia G. Practical conclusions for the future of malaria control. In: The Garki Project.Research on the epidemiology and control of malaria in the Sudan savanna of West Africa. Geneva: World Health Organization, 1980: 289–311.

9 Dietz K. Mathematical models for transmission and control of malaria. In: Wernsdorfer WH, McGregor I, eds. Malaria: principles and practice of malariology. Edinburgh: Churchill Livingstone, 1988: 1091–133.

10 Dietz K, Molineaux L, Thomas A. A malaria model tested in the African savannah. *Bull World Health Organ* 1974; **50:** 347–57.

11 Fine PE. Superinfection: a problem in formulating a problem (an historical critique of Macdonald's theory). *Trop Dis Bull* 1975; **72:** 565–71.

12 Aron JL. Mathematical modeling of immunity to malaria. *Math Biosci* 1988; **90:** 385–96.

13 Smith DL, McKenzie FE. Statics and dynamics of malaria infection in anopheles mosquitoes. *Malar J* 2004; **3:** 13.

14 Smith DL, McKenzie FE, Snow RW, Hay SI. Revisiting the basic reproductive number for malaria and its implications for malaria control. *PLoS Biol* 2007; **5:** e42.

15 Smith DL, Dushoff J, Snow RW, Hay SI. The entomological inoculation rate and *Plasmodium falciparum* infection in African children. *Nature* 2005; **438:** 492–95.

16 Ross R. The prevention of malaria. London: John Murray, 1911.

17 Ross R. An application of the theory of probabilities to the study of a priori pathometry. Part I. *Proc R Soc Lond A Math Phys Sci* 1916; **92:** 204–30.

18 Ross R, Hudson HP. An application of the theory of probabilities to the study of a priori pathometry. Part II. *Proc R Soc Lond A Math Phys Sci* 1917; **93:** 212–25.

19 Ross R, Hudson HP. An application of the theory of probabilities to the study of a priori pathometry. Part III. *Proc R Soc Lond A Math Phys Sci* 1917; **93:** 225–40.

20 Molineaux L, Muir DA, Spencer HC, Wernsdorfer WH. The epidemiology of malaria and its measurement. In: Wernsdorfer WH, McGregor I, eds. Malaria: principles and practice of malariology. Edinburgh: Churchill Livingstone, 1988: 999–1089.

21 Hay SI, Guerra CA, Tatem AJ, Atkinson PM, Snow RW. Urbanization, malaria transmission and disease burden in Africa. *Nat Rev Microbiol* 2005; **3:** 81–90.

22 Hay SI, Rogers DJ, Toomer JF, Snow RW. Annual *Plasmodium falciparum* entomological inoculation rates (EIR) across Africa: literature survey, internet access and review. *Trans R Soc Trop Med Hyg* 2000; **94:** 113–27.

23 Metselaar D, Van Thiel PH. Classification of malaria. *Trop Geogr Med* 1959; **11:** 157–61.

24 Beier JC, Killeen GF, Githure JI. Short report: entomologic inoculation rates and *Plasmodium falciparum* malaria prevalence in Africa. *Am J Trop Med Hyg* 1999; **61:** 109–13.

25 Macdonald G, Göeckel GW. The malaria parasite rate and interruption of transmission. *Bull World Health Organ* 1964; **31:** 365–77.

26 Dietz K. The estimation of the basic reproduction number for infectious diseases. *Stat Methods Med Res* 1993; **2:** 23–41.

27 Barat LM. Four malaria success stories: how malaria burden was successfully reduced in Brazil, Eritrea, India, and Vietnam. *Am J Trop Med Hyg* 2006; **74:** 12–16.

28 Nájera JA, Liese BH, Hammer J. Malaria. New patterns and perspectives. World Bank Technical Paper Number 183. Washington, DC: The International Bank for Reconstruction and Development (The World Bank), 1992.

29 Hay SI, Snow RW. The Malaria Atlas Project: developing global maps of malaria risk. *PLoS Med* 2006; **3:** e473.

30 Shiff C. Integrated approach to malaria control. *Clin Microbiol Rev* 2002; **15:** 278–93.

31 Feachem RG, Sabot OJ. Global malaria control in the 21st century: a historic but fleeting opportunity. *JAMA* 2007; **297:** 2281–84.

32 Global Fund. The Global Fund. Who we are what we do. Geneva: The Global Fund to Fight AIDS, Tuberculosis and Malaria, 2007.

33 Global Fund. An evolving partnership: the Global Fund and civil society in the fight against AIDS, tuberculosis and malaria. Geneva: the Global Fund to Fight AIDS, Tuberculosis and Malaria, 2007.

34 WHO. Global strategic plan. Roll Back Malaria 2005–2015. Geneva: World Health Organization, 2005.

35 Killeen GF, Smith TA, Ferguson HM, et al. Preventing childhood malaria in Africa by protecting adults from mosquitoes with insecticide-treated nets. *PLoS Med* 2007; **4:** e229.

36 Hill J, Lines J, Rowland M. Insecticide-treated nets. *Adv Parasitol* 2006; **61:** 77–128.

37 Lengeler C. Insecticide-treated bed nets and curtains for preventing malaria. *Cochrane Database Syst Rev* 2004; **2:** CD000363.

38 Le Menach A, Takala S, McKenzie FE, et al. An elaborated feeding cycle model for reductions in vectorial capacity of night-biting mosquitoes by insecticide-treated nets. *Malar J* 2007; **6:** 10.

39 Curtis CF, Maxwell CA, Finch RJ, Njunwa KJ. A comparison of use of a pyrethroid either for house spraying or for bednet treatment against malaria vectors. *Trop Med Int Health* 1998; **3:** 619–31.

40 Curtis CF, Mnzava AE. Comparison of house spraying and insecticide-treated nets for malaria control. *Bull World Health Organ* 2000; **78:** 1389–400.

41 Draper CC, Smith A. Malaria in the Pare area of Tanganyika. Part II. Effects of three years' spraying of huts with dieldrin. *Trans R Soc Trop Med Hyg* 1960; **54:** 342–57.

42 Foll CV, Pant CP, Lietaert PE. A large-scale field trial with Dichlorvos as a residual fumigant insecticide in northern Nigeria. *Bull World Health Organ* 1965; **32:** 531–50.

43 Molineaux L, Gramiccia G. Parasitology. In: The Garki Project. Research on the epidemiology and control of malaria in the Sudan savanna of West Africa. Geneva: World Health Organization, 1980: 109–72.

44 Smith A, Draper CC. Malaria in the Taveta area of Kenya and Tanganyika. Part II. Results after three and a half years' treatment of huts with dieldrin. *East Afr Med J* 1959; **36:** 629–43.
